# Revolutionizing crop disease detection with computational deep learning: a comprehensive review

**DOI:** 10.1007/s10661-024-12454-z

**Published:** 2024-02-24

**Authors:** Habiba N. Ngugi, Absalom E. Ezugwu, Andronicus A. Akinyelu, Laith Abualigah

**Affiliations:** 1 School of Mathematics, Statistics, and Computer Science, University of KwaZulu-Natal, King Edward Avenue, Pietermaritzburg, KwaZulu-Natal 3201 South Africa; 2 Unit for Data Science and Computing, North-West University, 11 Hoffman Street, Potchefstroom, 2520 South Africa; 3 Department of Computer Science and Informatics, University of the Free State, Bloemfontein, South Africa; 4 Artificial Intelligence and Sensing Technologies (AIST) Research Center, University of Tabuk, Tabuk, 71491 Saudi Arabia; 5 Computer Science Department, Al al-Bayt University, Mafraq, 25113 Jordan; 6 Hourani Center for Applied Scientific Research, Al-Ahliyya Amman University, Amman, 19328 Jordan; 7 MEU Research Unit, Middle East University, Amman, 11831 Jordan; 8 Department of Electrical and Computer Engineering, Lebanese American University, Byblos, 13-5053 Lebanon; 9 School of Engineering and Technology, Sunway University Malaysia, Petaling Jaya, 27500 Malaysia; 10 Applied Science Research Center, Applied Science Private University, Amman, 11931 Jordan; 11 College of Engineering, Yuan Ze University, Taoyuan, Taiwan

**Keywords:** Plant disease detection, Severity estimation, Deep learning, Machine learning

## Abstract

Digital image processing has witnessed a significant transformation, owing to the adoption of deep learning (DL) algorithms, which have proven to be vastly superior to conventional methods for crop detection. These DL algorithms have recently found successful applications across various domains, translating input data, such as images of afflicted plants, into valuable insights, like the identification of specific crop diseases. This innovation has spurred the development of cutting-edge techniques for early detection and diagnosis of crop diseases, leveraging tools such as convolutional neural networks (CNN), K-nearest neighbour (KNN), support vector machines (SVM), and artificial neural networks (ANN). This paper offers an all-encompassing exploration of the contemporary literature on methods for diagnosing, categorizing, and gauging the severity of crop diseases. The review examines the performance analysis of the latest machine learning (ML) and DL techniques outlined in these studies. It also scrutinizes the methodologies and datasets and outlines the prevalent recommendations and identified gaps within different research investigations. As a conclusion, the review offers insights into potential solutions and outlines the direction for future research in this field. The review underscores that while most studies have concentrated on traditional ML algorithms and CNN, there has been a noticeable dearth of focus on emerging DL algorithms like capsule neural networks and vision transformers. Furthermore, it sheds light on the fact that several datasets employed for training and evaluating DL models have been tailored to suit specific crop types, emphasizing the pressing need for a comprehensive and expansive image dataset encompassing a wider array of crop varieties. Moreover, the survey draws attention to the prevailing trend where the majority of research endeavours have concentrated on individual plant diseases, ML, or DL algorithms. In light of this, it advocates for the development of a unified framework that harnesses an ensemble of ML and DL algorithms to address the complexities of multiple plant diseases effectively.

## Introduction

Agriculture has been extensively employed as a means of encompassing many techniques including the cultivation of crops and the rearing of domesticated animals, all aimed at ensuring the long-term sustenance of the human population through food production (Bock et al., 2020). The marketing, processing, and distribution of agricultural products have become increasingly prominent in many countries as they heavily depend on the diverse yields of the agricultural sector. Agriculture, particularly in terms of agricultural yields, has been widely recognized as a fundamental requirement for economic growth in countries heavily reliant on it. According to pertinent reports (Skendžić et al., 2021), there is an annual escalation in the loss of crop yields and/or their quality as a result of crop illnesses and pest infestations. The exacerbation of the ecological population amplifies the factors contributing to the notable increase in the appearance of agricultural diseases in crops.

Moreover, crop complaint discovery, bracket, and inflexibility have long been an essential area to focus on regarding general agrarian practices, particularly crop products with relation to elements impacting crop output and quality (Rois-Díaz et al., 2018). Ineffective disease control, pollution, and other harmful effects are the result of farmers spending billions of dollars on disease management, frequently with little technical support (Tudi et al., 2021). In addition to wreaking havoc on natural ecosystems, plant diseases can exacerbate environmental issues caused by habitat loss and bad land management. Due to the crop’s significant financial contribution to the global economy, it is vital to monitor the crop’s health throughout its growth cycle by utilizing technology that offers more effective techniques to find crop-specific illnesses brought on by opportunistic pathogens.

There is a clear need for efficient and accurate disease detection systems in order to enhance crop yields. To address this demand, technological advancements such as the integration of Artificial Intelligence (AI) have been employed for the purpose of detecting, classifying, and estimating the severity of crop diseases (Chouhan, Kaul, & Singh, 2019a; Chouhan, Kaul, & Singh, 2019c; Chouhan, Kaul, & Sinzlr, 2019). The existence of AI is a topic of significant interconnection with factors such as the accessibility of food, the environment, resilience, and climate variability, making it a crucial and influential instrument. Researchers have offered multiple methods based on various aspects and performance of algorithm implementations in machine learning, specifically focusing on the algorithms themselves. The primary purpose is to achieve accuracy and efficient performance, which would provide prompt assistance in managing sick crops. This capability has been facilitated through the utilization of computer-assisted analytical tools, which can be accessible via a smartphone. Consequently, farmers are able to receive expert information at a reduced cost, as demonstrated by Fenu and Malloci (2021) and Dehnen-Schmutz et al. (2016).

Deep learning (DL) has made a substantial impact on the field of computer vision by enabling computers to do very precise tasks such as object detection, image categorization, restoration, and segmentation. More so, techniques that are grounded in deep learning context aim to replicate the functional capabilities of the human brain. In the absence of human expertise, Khan et al. (2021b) demonstrate that CNN, as an example, has the capability to autonomously discern the most optimal key elements from input samples. It has demonstrated exceptional accuracy in the domain of agriculture and has effectively adjusted to many applications. This manuscript presents the current state-of-the-art of ML-based and DL-based crop detection, classification, and severity estimation techniques. This paper also highlights recent achievements, relevant research challenges, and future research directions. This review is different from the existing surveys in the following ways:

i. Most review papers considered either ML or DL techniques, but our study encompasses both ML and DL-based approaches for crop disease detection.
ii. The majority of review papers examined either crop detection or classification strategies, with a limited number of publications specifically addressing severity estimation techniques. This study provides an overview of three distinct strategies: crop disease detection, crop disease classification, and severity estimating techniques.
iii. Several review papers lacked a thorough examination, performance evaluation, and presentation of significant discoveries. This paper provides a thorough examination and performance evaluation of crop disease strategies based on deep learning (DL) and machine learning (ML). This paper also outlines certain gaps, recent advancements, pertinent research challenges, and prospective avenues for future research. The provided information can serve as a foundation for further scholarly investigation.

Moreover, it is worth noting that the present paper is dedicated to exploring the latest research trends in the area of plant disease detection, classification, and severity assessment. To provide further context and clarity, the technical contributions of this study are detailed below:

- A comprehensive survey of current literature encompassing methods for the diagnosis, classification, and assessment of the severity of crop diseases.
- In-depth discussions of diverse performance analysis approaches applied to the most recent machine learning (ML) and deep learning (DL) techniques presented in scholarly works.
- An overview of recent research endeavours focusing on publicly accessible datasets related to plant diseases.

## Preliminary concepts

This section presents some fundamental concepts that are crucial for a proper comprehension of this literature survey.

### Crop disease detection

Crop disease detection refers to the evaluation of disease occurrence, intensity, and impacts (Abdu et al., 2020). Crop disease can be characterized as a dynamic phenomenon that disrupts the normal functioning of plants and hinders their growth during a specific time frame (Abdu et al., 2020). The identification of crop illnesses involves the utilization of machine vision techniques to acquire images, which are subsequently subjected to analysis in order to ascertain the presence or absence of pathogens within the crop (Liu & Wang, 2021).

### Crop disease classification

Barure et al. (2020) describe crop disease classification as the process of categorizing each subject under study into various groups. This definition may be used to analyze an object’s measurements and determine which group the leaf falls to; the process of classifying crop diseases involves taking information from infected plant leaves and determining the differences in colour between diseased and healthy crop leaves (Halder et al. (2018)). It is either supervised or unsupervised and uses a variety of techniques, including colour information, image borders, or image segmentation.

### Crop disease severity estimation

Crop disease severity is defined as the portion or percentage of visible diseased crop tissue relative to the total amount of crop tissue. In contrast to the total number of crops affected, it is a continuous variable with two binary values of zeros and ones that represent the degree of crop tissue quality. Bock (2022) identifies it as the crop unit of quantity showing perceptible disease symptoms, which is frequently expressed as a percentage. According to Bock (2022) and Wu et al. (2019), in order to predict production and claim its control, crop disease severity is regarded as a crucial metric for assessing the disease’s level of infection. Bock (2022) elaborates further that plant disease severity is the quantification, mainly the intensity of disease symptoms on individual units, and thus is the basis for a plethora of research and applied purposes in plant pathology and related disciplines.

### Machine learning and deep learning

It has been shown that algorithms used in artificial intelligence (AI), such as ML and DL algorithms, give precise forecasts. DL, a vital element of data science, uses nonlinear algorithms that are layered in complication and abstraction in contrast to ML that uses linear algorithms. This involves predictive analysis and statistics, which mimic how people acquire some knowledge. Abdu et al. (2020) discussed that precision agriculture has seen a huge growth in the usage of image-based procedures using ML-based methodologies, which was ascribed to the convenience of higher-quality measurements together with modern algorithms and an expanded ability to fuse different sources of pictures.

**Fig. 1:**
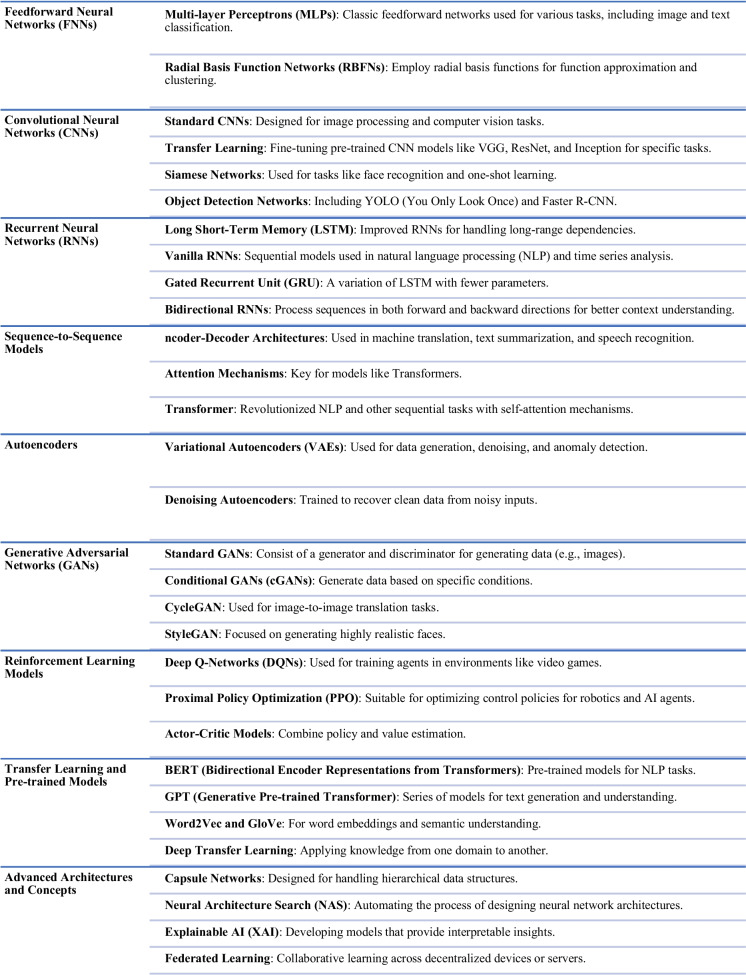
A taxonomy of various deep learning models

**Fig. 2:**
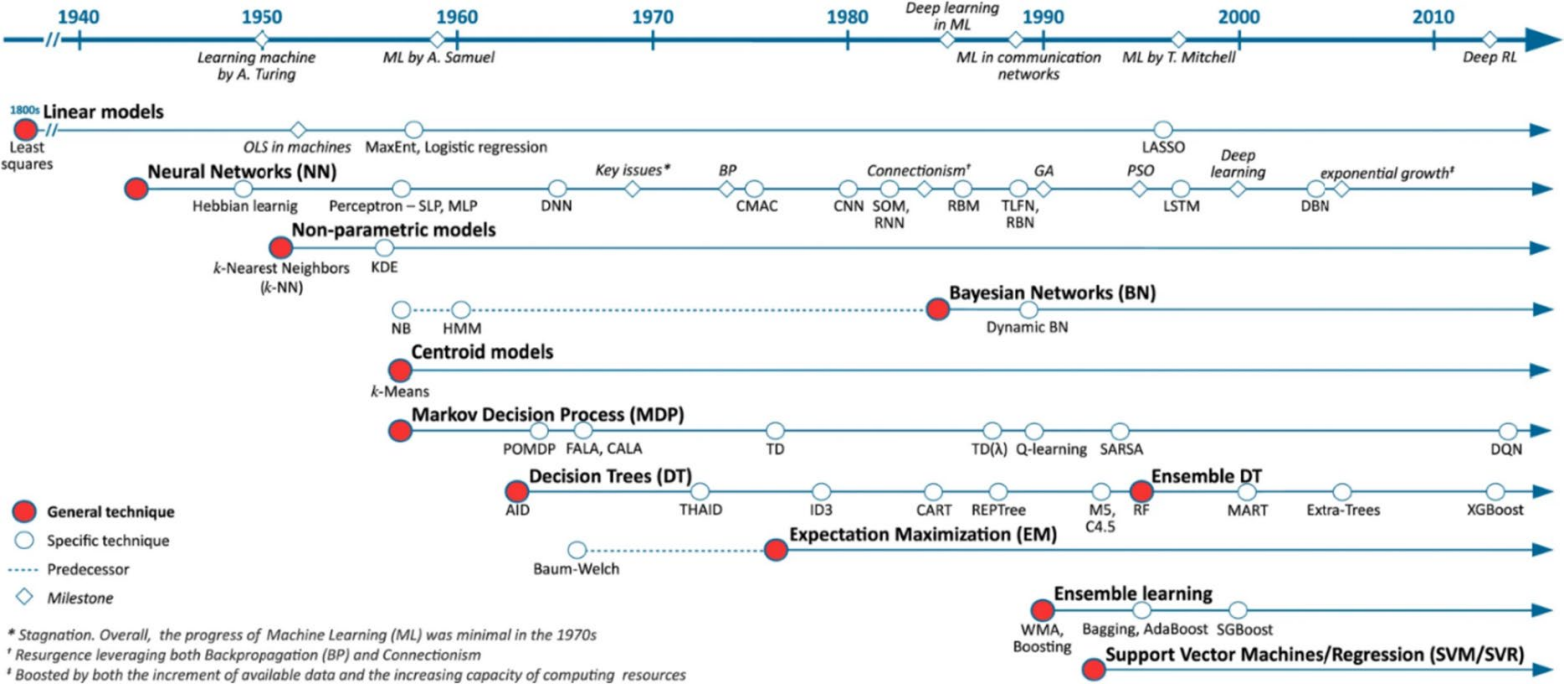
Summary of the evolution of classical ML models (Boutaba et al., 2018)

A stack of convolutional is employed in the DL methodological classifier that includes numerous levels of information processing stages. These layers are ultimately used for feature learning, analysis, and pattern classification. Examples of these classifiers are as follows: recurrent neural networks (RNNs), deep neural networks (DNNs), and convolutional neural networks (CNN). The most prominent CNN assesses input data that have been labelled and examines at the co-relation feature attributes that are taken from an image’s region of interest during training, which have shown great result for classifying objects and images. By giving computers the highest level of precision achievable for object identification, image categorization, restoration, and segmentation, DL has significantly improved computer vision (Khan et al. (2021b)). Figure 1 presents the taxonomy of several DL models from inception to date, while Fig. 2 gives the summary of the evolution of classical machine learning models.

### Review scope

Our search identified 155 relevant articles of which 110 were original publications, 20 conference proceedings, 15 review articles, and ten additional items (including editorials and agricultural organizations). DL methods received more attention in the publications than ML algorithms due to their sophisticated features that provide amazing results of performance metrics analysis, particularly accuracy in crop disease detection and classification. Crop disease severity estimation, in particular, contains few articles because it is a new topic of study. This is ascertained from the few number of articles available. This necessitates a greater focus on crop disease severity estimation research in future.

## Review of related work

This section presents a review of ML-based and DL-based techniques for crop disease classification, detection, and severity estimation. Firstly, a review of ML-based techniques for plant disease detection, classification, and severity estimation is presented. Further, a review of DL-based techniques is presented. Table 1 offers a concise summary of prior review research in comparison to the scope of the present work, emphasizing a discussion on the strengths and limitations of the existing studies.

**Table 1:** A concise summary comparing the previous studies and current review work

References	Coverage areas	Strengths	Limitations
Ngugi et al. (2021)	The review presented a thorough examination of recent research conducted in the field of identifying crop pests and diseases through the application of image processing and machine learning methods	Provides a comprehensive review of recent research on automated leaf disease recognition using image processing techniques, focusing on RGB images Summarizes important studies on different crops/diseases and compares techniques used for pre-processing, feature extraction, and classification Discusses the transition from using hand-crafted features and shallow classifiers to deep learning approaches like CNNs Presents experimental results comparing performance of 10 CNN architectures on a leaf disease dataset Identifies gaps in current research needed to develop practical systems for field use	The review focuses only on RGB images and does not cover other imaging modalities like hyperspectral Does not provide details on the datasets and codes used in the different studies, limiting reproducibility Performance comparisons of previous works are difficult since different datasets and evaluation metrics were used Discussion of deep learning approaches is relatively brief compared to classical methods Does not provide an independent evaluation of the different techniques on a standardized dataset Does not propose a comprehensive solution integrating recent advances to address current gaps
Golhani et al. (2018)	The paper reviewed advanced neural network techniques available to process hyperspectral data, with a special emphasis on plant disease detection	Provides a comprehensive review of neural network techniques for analyzing hyperspectral data to detect plant diseases. It covers the different types of neural networks, models, classifiers, and applications Discusses how neural networks can help develop spectral disease indexes from hyperspectral data to simplify and expedite disease detection Highlights studies that have successfully applied neural networks combined with hyperspectral imaging or spectroscopy for early detection of various plant diseases Discusses the advantages of neural networks for hyperspectral data analysis including their ability to model complex nonlinear relationships and perform pattern recognition without explicit rules	Only provides a brief overview of two studies applying neural networks to rice disease detection, more in-depth analysis could have been provided Does not evaluate or compare the performance of different neural network techniques for disease detection Does not propose any new spectral disease indexes developed using neural networks on hyperspectral data Future trends section only briefly mentions deep learning and does not discuss how it could advance disease detection using hyperspectral data Does not discuss challenges in applying neural networks to real-world disease detection problems at large scales
Li et al. (2021)	The review discusses the advancements in deep learning technology for identifying crop leaf diseases over recent years. The paper explores current trends and challenges in plant leaf disease detection, focusing on the application of deep learning and advanced imaging techniques	Provides a comprehensive review of recent research on plant disease detection and classification using deep learning techniques. It discusses various state-of-the-art deep learning architectures that have been applied Discusses emerging techniques like data augmentation using generative adversarial networks (GANs) for handling limited plant disease datasets Reviews applications of hyperspectral imaging and newer visualization techniques used along with deep learning models to better understand disease detection mechanisms Identifies current challenges like small sample detection and discusses potential directions to address them	Does not provide a quantitative comparison of different deep learning models on common benchmark datasets Discussion of visualization techniques is at a high level without illustrating specific examples Scope for more discussion on integrating deep learning techniques with domain knowledge from plant pathology for disease symptom-level classification Future research directions could be proposed more concretely based on gaps identified in the review Being a review article, it does not present any new empirical results or methodology to address open challenges
Liu and Wang (2021)	The study delineates recent research on plant disease and pest detection using deep learning, focusing on three aspects: classification networks, detection networks, and segmentation networks. The advantages and disadvantages of each method are also summarized	Provides a comprehensive review of plant disease and pest detection methods based on deep learning from 2014 to 2020. This gives researchers an overview of progress in this area Clearly categorizes and explains different deep learning approaches according to network structure (classification, detection, segmentation networks). This organization helps the reader understand the different methodologies Discusses and compares popular deep learning models used for plant disease or pest detection such as AlexNet, ResNet, and Inception. This demonstrates the state-of-the-art approaches. Summarizes commonly used datasets and compares performance of existing studies. This provides useful benchmarks for evaluating new research	Only considers research published up to 2020, so does not include the most recent developments in 2023 and beyond Performance comparisons are limited and high-level due to varying experimental settings across different studies. More rigorous evaluation may be needed Discussion of challenges is brief and does not offer many specific solutions. Deeper examination of open problems could help guide future research Lacks discussion of dataset biases, data imbalances, and other model vulnerabilities—factors important for real-world deployment of these techniques Organization focuses on network structures but does not consider other axes like problem type (classification vs segmentation) which may be useful
Dhaka et al. (2021)	The review conducted a comprehensive examination of the current literature on the utilization of deep convolutional neural networks for predicting plant diseases based on leaf images	Provides a comprehensive survey of deep convolutional neural network (DCNN) techniques for plant leaf disease detection from images. The authors in their study compared various pre-processing methods, CNN models, optimization techniques, and frameworks Highlights key advances in the field from 2015 to 2021 when deep learning methods gained popularity over traditional machine learning The authors’ work discusses important considerations like dataset collection and annotation, model architectures, training processes, and performance evaluation metrics The study summarizes advantages and limitations of different approaches to help researchers select suitable methods	Does not empirically evaluate the performance of the discussed methods on common benchmark datasets Mostly focuses on previous work and does not propose new contributions to further advance the field Comparison of techniques is based on a limited literature review rather than experimental results Certain technical details of important models and techniques are not fully explained due to the survey format
This work	The current review examines the performance analysis of the latest ML and DL techniques as outlined in these studies. It also analyses the methodologies and datasets and outlines the prevalent recommendations, and identified gaps within different research investigations	The ongoing research effort provides a thorough literature survey that encompasses techniques for diagnosing, classifying, and assessing the severity of crop diseases Furthermore, the study explores various performance analysis approaches that have been applied to the latest ML and DL techniques in terms of crop disease diagnoses, classification, and severity assessment Additionally, the study examined recent research efforts, offering an overview of publicly accessible datasets related to plant diseases	The review predominantly concentrated on the performance analysis and evaluation of the latest ML and DL techniques. Consequently, it lacks an in-depth exploration of state-of-the-art classical ML model analysis, which could have been employed to address similar problems discussed in the present study

### Machine learning-based crop disease detection and classification techniques

Machine learning-based crop disease detection and classification techniques have gained significant attention in agriculture for early and accurate diagnosis of plant diseases. These techniques utilize various algorithms and data sources to help farmers and researchers identify, monitor, and manage crop diseases. Here are some common methods and approaches used in this field.

#### Crop disease detection using Random Forest

Maniyath et al. (2018) developed a crop disease detection model using Random Forest (RF) algorithm and Histogram of an Oriented Gradient (HOG). They used HOG to extract features from a labelled image dataset consisting of 160 images of papaya leaves. The extracted features are used to build the RF model. The model produced a classification accuracy of 70.14%. As noted by the authors, the accuracy can be improved by training the model on a larger number of images. The performance of the model can also be improved by training it on a combination of local and global image features, such as SIFT (Scale Invariant Feature Transform), SURF (Speed Up Robust Features), and DENSE along with BOVW (Bag of Visual Word).

#### Crop disease detection using image-based features, KNN, and GLCM

Gaikwad and Musande (2023) developed an advanced crop disease prediction technique using KNN, cetalatran optimization algorithm, and relief algorithm. The relief algorithm is used to select relevant features from high-resolution hyperspectral UV images by evaluating the strong dependencies and quality of attributes. In addition, it allocates a weight to each feature present in the training dataset and then selects the weighted features with the highest average relevance to crop disease prediction. The feature is measured using Boolean functions, integer numbers, or real values. The selected features are used to train the KNN classifier. The classifier is tuned using the cetalatran optimization algorithm. The cetalatran optimization algorithm consists of two optimization algorithms, namely Coyote optimization algorithm and Dolphin echolocation algorithm. The proposed method was evaluated, and it achieved a classification accuracy, sensitivity, and specificity of 89.12%, 93.39%, and 92.77%, respectively.

Harakannanavar et al. (2022) developed an algorithm based on ML and image processing (IP) to automatically detect leaf diseases. Histogram equalization and k-means clustering are used for quality maximization and segmentation thus enabling early stage of operation prediction of whether the leaf is healthy or unhealthy. DWT, PCA, and GLCM are used for feature extractions, and finally, SVM, KNN, and CNN are used to classify the features. The accuracy of the three ML used was given as 88%, 97%, and 99.6%, respectively, with CNN being the most recommended due to its highest accuracy.

Komala (2021) proposed an image processing and filter-based feature selection method that distinguishes and classifies crop diseases using k-means, GLCM, and KNN. k-means was used for image segmentation after being pre-processed to enhance its contrast. Feature extraction from the leaf image was done using GLCM. The extracted features were used to train a KNN classifier. The model achieved a performance accuracy of 95% compared to an accuracy of 88% without feature selection. The model is highly recommended due to its improved accurate results that contrast with other ML and image processing algorithms.

#### Crop disease detection and classification using SVM

Suresh (2023) designed an automated approach that would send unhealthy images to their database for ailment analysis, categorization, and remedy sent back to the producer. Artificial neural network (ANN), support vector machine (SVM), and transfer Learning (TL) were used for generation of plants that enhanced the accuracy of agricultural disease classification. ANN was used for recognition using its interconnected layers that can learn and process data. SVM was used for classification using hyperplanes and TL was reused for accuracy and efficiency improvement of the model since the dataset used of paddy leaves was fairly little. The dataset contained 2149 of both healthy and unhealthy paddy leaf images.

In another study, Ahmed and Yadav (2023) developed a predictive model by employing the backpropagation artificial neural network algorithm together with three ML and one feature selection techniques, namely SVM, k-means clustering, CNN, and GLCM. SVM was employed for the purpose of classification, while GLCM was utilized for feature extraction. k-means clustering was employed to identify diseases in infected crop images obtained from real-time leaf images. Additionally, CNN was employed for the training and fine-tuning processes. The prediction model underwent training using the PlantVillage dataset, and it achieved a precision rate of 99%.

Abdu et al. (2020) contrasted two well-known ML methods (AlexNet and SVM) for crop disease identification. The two models were trained on plant images from the PlantVillage dataset, and the two attained classification accuracy of 97% and 95%, respectively. The results showed that AlexNet outperformed SVM, making it the preferable choice for large-scale datasets. The study’s findings also demonstrate that SVM are well-suited for classification tasks involving small-scale datasets. The little misclassification seen in both models can be attributed to the finding that samples displaying initial symptoms of the late blight disease were the primary contributors.

Jangid (2023) designed and constructed a web-based application for diagnosing disease in rice leaves. They classified rice illnesses using VGG16. The model was trained using 4500 healthy and unhealthy rice leaf images from the Kaggle website. The model had a 90% accuracy rate.

Abdu et al. (2020) conducted a comparative analysis using the model implementation of two well-known ML and DL models (SVM, DL) for plant disease diagnosis. The two techniques, SVM and DL, were evaluated using typical parameters on a PlantVillage dataset of 2152 images of crop leaf disease using conventional settings. Among various ML classifiers, SVM was named the most preferred learning algorithm in crop disease classification. With DL, SVM is known for employing fewer datasets and is likely to have a trade-off between restricted modelling and representation power. RGB colour space was utilized to depict various illness severity estimation levels.

The GLCM technique was utilized to extract textural features from the ROI, and the colour moment was also employed to retrieve colour information. Architecture, processing capability, and training data amount were identified as three major challenges.

DL meta-architectures were used to perform a hard task of crop disease localization and classification for image-based plant disease identification (Hammad Saleem et al. (2020)). Tensor flow object detection framework was used to apply three DL meta-architectures: single shot multibox detector (SSD), faster region-based CNN, and region-based fully CNN. For plant species disease recognition, all DL models were trained and tested in a controlled dataset environment. The SSD model, which was trained using the Adam optimizer, has the greatest mean average precision of 73.07%. The proposed framework can as well be adopted for other agricultural applications. Table 2 presents an overview of crop disease detection and classification strategies that are based on machine learning techniques.

**Table 2:** A summary table on machine learning in disease detection and classification

Reference	Algorithm used	Results	Key contributions	Datasets
Maniyath et al. (2018)	Random Forest	Accuracy, 70.14%	The authors designed a RF-based technique for crop disease detection	Private (160) papaya leaves
Gaikwad and Musande (2023)	Cetalatran-optimized deep KNN in multispectral imaging	Accuracy, 89.12%; sensitivity, 93.39%; and specificity, 92.77%	Developed an advanced crop disease prediction technique using cetalatran-optimization algorithm, deep KNN, and relief algorithm	Private
Harakannanavar et al. (2022)	SVM, KNN, and CNN	Accuracy, 88%, 97%, and 99.6%, respectively	Developed an algorithm based on ML and image processing (IP) to automatically detect leaf diseases	PlantVillage tomato leaves
Komala (2021)	k-means, KNN, and GLCM	Accuracy, 90% k-means and KNN, 95%	The authors proposed a plant leaf disease detection and classification technique using k-means	Private, carrots leaves
Suresh (2023)	ANN, SVM with TL		Designed an automated application that would send unhealthy images to their database for analysis of its ailment, categorize it, and send back the remedy to the producer	Private (2149) paddy
Ahmed and Yadav (2023)	SVM, GLCM, k-means	Accuracy, 99%.	Developed a crop disease prediction model using back propagation ANN, SVM, GLCM, and k-mean algorithm	PlantVillage (8350), 12 crop species
Abdu et al. (2020)	SVM and deep convolution neural network (DCNN)	Accuracy, 97% DCNN, 98.92%	Developed a crop disease detection and classification method using SVM and DL algorithms	PlantVillage (2152) potato
Anjna et al. (2020)	KNN, GLCM, SVM	Accuracy, 100%	Designed a detection and classification technique using k-means, GLCM, and SVM	Private (70) capsicum
Barure et al. (2020)	CNN	Accuracy, 94%	Designed a decision-making system using CNN image content characterization and supervised classifier	PlantVillage (7000), Private (15,000), 4 crop species
Jangid (2023)	VGG16	Accuracy, 90%	Created and implemented a web-based application that would identify diseases that would affect rice leaf	Kaggle (4500) rice
Maniyath et al. (2018)	RF	Accuracy, 70%	RF classifier was used for crop disease classification, HOG feature descriptor	Private (160) papaya
Komala (2021)	KNN, GLCM	Accuracy, 95% with optimal feature selection, 90% without	The authors proposed an image processing, filter-based feature selection technique for crop disease distinction and classification	-
Abdu et al. (2020)	RBF kernel classifier was implemented, SVM a linear classifier	Accuracy, 95.8%	The authors implemented SVM and DL for crop disease detection	Private
Hammad Saleem et al. (2020)	3DL meta-architecture	Accuracy, 73.03%	The authors performed localization and classification of crop disease in leaves using three DL meta-architectures (SSD, RCNN, and RFCNN) in several plant species infected by virus, fungi, and bacterial infections	PlantVillage

#### Crop disease detection using k-means clustering, KNN, and GLCM

Anjna et al. (2020) developed a technique for automated crop leaf identification and classification using k-means clustering, SVM, and KNN. The k-means clustering technique was used to identify and extract a sick plant leaf, which was then subjected to feature extraction using the GLCM method. The SVM and K-nearest neighbours (KNN) were used for classification. The methodology was assessed using the private dataset of 62 images both healthy and unhealthy, resulting in a classification accuracy of 100%. This proposed technique intends to assist farmers in identifying plant diseases, providing them with necessary data for informed decision-making, and facilitating the rapid selection of effective remedies.

Komala (2021) proposed an image processing and filter-based feature selection technique for distinguishing and classifying diseased images. KNN classifier with k-means clustering, GLCM feature extraction, and KNN were employed in the methodology. The proposed technique sought to forecast leaf disease using image processing, boost contrast and segmentation using the k-means algorithm, and texture feature extraction using GLCM. Using feature selection, the leaves’ feature set can be made more controlled, allowing for the execution of upgraded processes. It also helps with cost training reduction, accurate learning of the learning algorithm, and accurate classification, which is the desired final output. The optimal feature selection strategy improved disease detection on plant leaves from 88.52% accuracy without feature selection to 95.21% increasing the effectiveness of KNN identification.

#### Crop disease classification using Random Forest

Maniyath et al. (2018) developed a RF-based technique for identifying between healthy and diseased leaves. The technique was trained on a private dataset consisting of 160 images of papaya leaves and it produced a classification accuracy of 70.14%. The performance of the model can be improved when trained on a dataset with more images. The model’s performance can also be improved if it is trained on a combination of local and global features, such as Scale Invariant Features Transformation (SIFT), Speed up Robust Features (SURF) and DENSE, and h Bag of Visual Word (BOVW). RF algorithm is flexible in nature and it is more accurate if trained on a small- and medium-scale image dataset.

### Deep learning-based crop disease diagnosis techniques

Deep learning-based crop disease diagnosis techniques have gained significant attention in recent years due to their potential to revolutionize agriculture by enabling the early and accurate detection of plant diseases. These techniques leverage deep neural networks and large datasets of images to automatically identify and classify crop diseases. In the following subsections, some of the key DL-based crop disease classification, detection, and severity estimation strategies are presented and discussed accordingly.

#### Deep learning-based technique for crop disease severity estimation

Deep learning-based techniques for crop disease severity estimation have the advantage of being able to handle complex and dynamic disease patterns. They can be applied in precision agriculture to optimize treatment strategies, reduce the use of pesticides, and enhance crop management practices. These techniques are particularly valuable in detecting and assessing the impact of diseases at an early stage, which is crucial for preventing extensive crop losses. The subsequent subsections provide an overview of the different DL-based strategies for estimating crop disease severity.

#### Crop disease severity estimation using CNN

Shi et al. (2023) reported that there are relatively few studies on disease severity assessment; thus, their aim is to tracing prevailing views of existing studies in order to provide its grading criteria. They also addressed the main obstacles that CNN-based plant disease severity assessment systems confront in practical applications and gave plausible research ideas and potential ways to address them. Additionally, they discussed core challenges faced by CNN-based crop disease severity assessment methods in practical applications and provided feasible research ideas and possible solutions to address these challenges. This was accomplished by presenting a review of 16 studies and providing a comprehensive comparative analysis of different CNN-based plant disease severity assessment frameworks, improved CNN architectures, and CNN-based segmentation with respect to the various CNN architectures used in the study. The survey showed that CNN-based segmentation theory has been widely employed in disease severity estimation, with the basic goal of assigning appropriate labels to each pixel in order to calculate the percentage of diseased areas required for disease severity assessment.

Fenu and Malloci (2021) developed a DL-based for crop disease severity estimation. They categorized three diseases and six severity levels. Moreover, they used five pre-trained CNN architectures as feature extractors, namely VGG16, VGG19, ResNet50, Inception V3, MobileNetV2, and EfficientNetB0 which emerged as the best with an accuracy of 78.31%. This was accomplished using computational experiments on the DiaMOS plant dataset for model validation. The results showed that the trained model is robust in automatically extracting disease leaf detection characteristics using a multi-task learning strategy.

#### Crop disease severity estimation using ResNet50

Wspanialy and Moussa (2020) developed a novel computer vision system to automatically recognize a number of diseases, find diseases that had not been observed before, and estimate the severity of each leaf. The proposed method focused on recognizing generic features that are associated with diseases regardless of disease type allowing for the detection of previously unseen instances using ResNet50 to detect and U-Net DL learning architecture to estimate disease severity. They also emphasized the significance of assessing disease severity or incidence. While severity is a more accurate estimate of the ratio of afflicted area based on leaf coverage, incidence is a shorter expression that refers to disease frequency measurement at the individual plant level.

However, the ResNet50 model was trained on the PlantVillage dataset to recognize visual properties common to all objects, such as corners, lines, and other patterns, and its weights were used as the foundation for transfer learning to identify diseases. The dataset consists of 16,415 unhealthy images which exhibited 9 different conditions and 1590 healthy tomato images. ReLu was used as the model’s activation function, and it was trained using a sophisticated gradient descent optimizer with a weighted loss to account for the unequal sample distribution in the training dataset. U-Net DL architecture was used to estimate tomato leaf severity on a hybrid DL model.

The pixels in an image were divided into three categories: health, sickness, and backdrop. The absolute difference between real and anticipated severity values (severity error) was used to assess the severity estimate’s performance, whereby it only considered how accurate the severity estimation was without considering if the specific areas were marked correctly. The performance metrics of mean severity error on the bacterial spot was 90% as compared to the mean Jaccard index of 40.8% thus the previous being highly recommended.

#### Crop disease severity estimation using Inception V3

Pang (2020) aimed at assessing the performance of 6 different types of popular DL models and choosing the most suitable for HLB severity estimation detection thus provide a constructive advice for farmers. DC-generative adversarial networks (DC-GANS) were employed to increase the training dataset size by performing augmentation on the original dataset of 5406 to 14,056. DC-GANS was also used to investigate whether the augmented data are helpful to improve the learning performance of the selected suitable model.

A sweet orange dataset with three different severity levels of HLB infection (early, moderate, and severely infected stages) was generated based on the 5406 citrus leaf pictures with Huanglongbing (HLB) that were obtained from PV and CrowdAI. Among the 6 different and popular DL models namely AlexNet, VGG, ResNet, Inception V3, SqueezeNet, and DenseNet were trained to detect severity under identical settings. Inception V3 was termed the best with an accuracy of 74.38% with epochs = 60 for severity identification due to its superior computational efficiency and low number of parameters. The deep convolutional generative adversarial networks (DCGANs) have demonstrated an outstanding accuracy of 92.60%, outperforming the Inception v3 model trained with the original dataset by around 20%.

#### Crop disease severity estimation using VGG16

Wang et al. (2017) developed a DL model for image-based automatic diagnosis of plant disease severity estimation, inspired by a DL breakthrough in image-based plant disease recognition. This was accomplished by training CNNs of varying depths from scratch and fine-tuning four cutting-edge deep models: VGG16, VGG19, Inception V3, and ResNet50. PlantVillage dataset of 2086 healthy and unwell apple leaf images was annotated for the training. When these networks were compared, it was discovered that fine-tuning on pre-trained deep models can considerably enhance performance on little amounts of data.

Additionally, VGG (16 and 19) demonstrated a significant improvement over previous setups by employing a design with relatively small convolution filters. GoogLeNet inception architecture combines the network-in-network approach and the strategy of dealing with different scales by deploying a series of filters of varying sizes. Inception V3, an improved architecture, features a low parameter count and high computing efficiency. ResNet is built using stacks of residual building blocks, each of which is composed of a number of convolutional layers connected by a skip link. Skip connections execute identity mappings, making optimization easier; each stacked layer fits a residual mapping. The VGG16 model produced the greatest results, with an accuracy of 90.4% on the test set, with deep network training accuracies approaching 100% and triggering the early halting. Because DL is data-driven, training on more data will improve test accuracy even more. Deep Learning, as demonstrated by VGG16, is a new promising technology for totally autonomous plant disease severity categorization.

#### Crop disease severity estimation using a Real-Time Kinetic Global Positioning System

Clohessy et al. (2021) developed a file-based, high-throughput evaluation tool that quantifies the geographical distribution of disease symptoms over experimental peanut plots using a Real-Time Kinetic Global Positioning System (RTK GPS), consumer-grade cameras, a microcontroller, and open-source machine learning software. A field experiment was also planned to simulate various illness incidence and severity scenarios. The field experiment was recorded for two seasons in order to design and assess the technology. TL was employed on an existing CNN for training images to identify and quantify symptomatic, asymptomatic, and ground regions within plot-level imaging. Furthermore, the machine learning model assessed numerous images and associated them with particular experimental plots through the utilization of RTK-GPS data.

The CNN model trained to identify the symptom “stunning and smottling” generated sensitivity of 77% and specificity of 98% on the test set. The technique for high throughput disease severity assessment in peanut in field conditions was successfully implemented. Table 3 summarizes approaches for estimating crop disease severity using DL.

**Table 3:** Summary methods for DL-based crop disease severity estimation

Reference	Algorithm used	Results	Key contributions	Datasets
Shi et al. (2023)	**CNN**	**An estimate of 90.4% accuracy for the most efficient DL models**	Addressed the main obstacles that CNN-based plant disease severity assessment systems confront in practical applications and gave plausible research ideas and potential ways to address them.	PlantVillage
Fenu and Malloci (2021)	EfficientNetB0	Accuracy, 78.31%	Categorized 3 diseases and 6 severity levels with 5 pre-trained CNN architectures that were used as feature extractors	PlantVillage
Wspanialy and Moussa (2020)	ResNet and U-Net	Accuracy, 98.7%	Built a novel computer vision system to automatically recognize a number of diseases, find diseases that had not been observed before and estimate the severity of each leaf	PlantVillage (18,005) tomato
Pang (2020)	Inception V3 DCGANs (Deep Convolutional Generative Adversarial Networks)	Accuracy, 74.38% with 60 epochs, 92.60% with GAN-based data augmentation	Aimed at assessing the performance of 6 different types of popular DL models and choosing the most suitable for HLB severity estimation detection thus provide a constructive advice for farmers	PlantVillage and CrowdAI (5406) sweet oranges leaves
Wang et al. (2017)	VGG16	Accuracy, 90.4%	Developed a DL model for image-based automatic diagnosis of plant disease severity estimation, inspired by a DL breakthrough in image-based plant disease recognition	PlantVillage (2086) apple leaves
Clohessy et al. (2021)	CNN	Sensitivity, 77%; specificity, 98%	Developed a file-based, high-throughput evaluation tool that quantifies the geographical distribution of disease symptoms over experimental peanut plots using a Real-Time Kinetic Global Positioning System (RTK GPS)	PlantVillage (4,704) peanuts

### Deep learning-based techniques for crop disease detection and classification

**Fig. 3:**
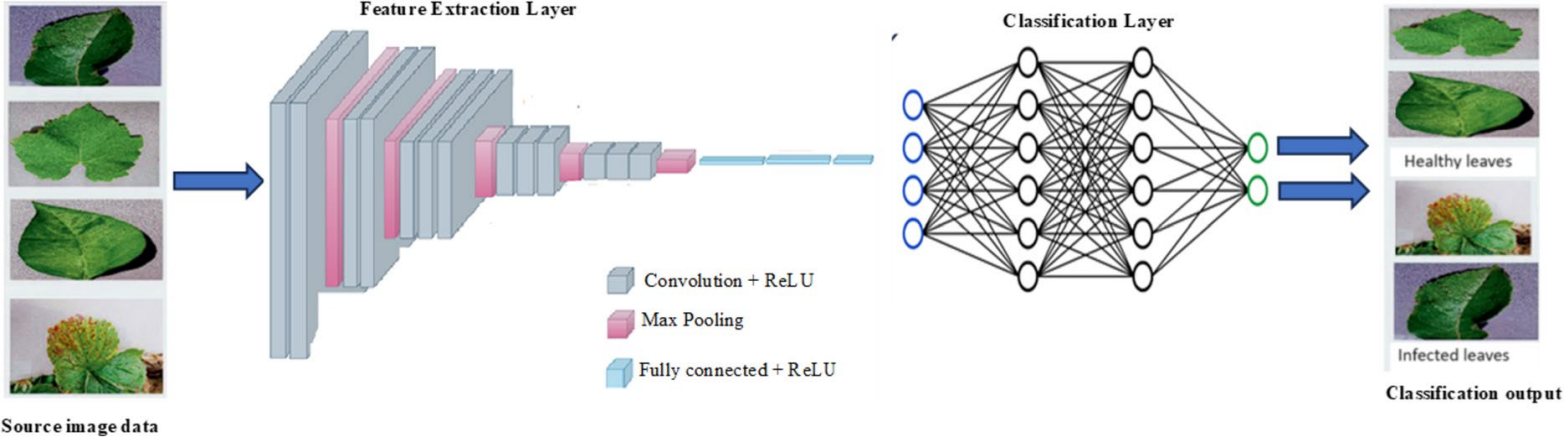
Generalized representation of DL-based crop disease detection and categorization model.

Deep learning-based techniques have significantly advanced the field of crop disease detection and classification. These approaches leverage neural networks to automatically identify and categorize diseases in plants, offering several benefits to agriculture. More so, DL-based techniques for crop disease detection and classification offer a promising path to improving crop health, increasing yields, and reducing the environmental impact of agriculture. This section discusses some DL-based crop disease detection and categorization strategies. In Fig. 3, the generalized DL-based crop disease detection and categorization strategies are presented.

#### Crop disease detection using a customized DL framework

Utilizing rice PlantVillage (21,198), Reddy et al. (2023) proposed a customized PDICNet framework/model for crop disease identification and classification. To obtain improved classification performance, ResNet50, modified red deer optimization algorithm (MRDOA), and DL-CNN classifier were used. ResNet50 is well recognized for segmentation operations and was thus used at the key point calculation level to create the deep features extraction of the images. MRDOA was used as a feature selection technique to generate smaller features and CNN classifier to produce optimized features in order to develop a classification model to predict illness from rice crop photos. The proposed model PDICNet achieved performance matrices of 99.73% accuracy, precision 99.83, recall 99.72%, and f-measure 99.78% with PlantVillage dataset. Additionally, performance metrics were done using the rice crop dataset of 400 images from Kaggle that resulted to 99.68% accuracy, 99.72 precision, 99.70% recall, and f-measure 99.71%. The proposed model PDICNet outperformed ResNet50 in terms of performance.

Kumbhar et al. (2019) developed a tool that leverages user-uploaded images to identify cotton leaf diseases by enhancing it to be a user-friendly web-based system for farmers. This was achieved by inputting unhealthy cotton leaf images for processing. CNN was then used to predict the disease with the highest risk of occurring and thereafter provide curative and preventative measures for the detected diseases. They used the private dataset of 513 both healthy and unhealthy hence achieving an accuracy of 80% on training and 89% accuracy on testing.

#### Crop disease detection using MobileNet

Gandhi et al. (2021) called for the DL incorporation to reduce crop loss due to diseases. They used DL methods such as MobileNetV2, a neural network architecture developed by Google, to push the boundaries of mobile visual identification for both object detection and classification. This network was chosen because it can achieve high accuracy while operating in a limited resource environment.

Siamese Network is a notion that can directly learn picture mappings, allowing it to generalize over fresh data and be helpful even with limited training data. The neural network model combined MobileNetV2 and Siamese networks to achieve accurate results for crop disease detection. The proposed method made used universal use of Android smartphones to install a neural network capable of detecting crop illness at an early stage, allowing effective measures to be performed before damage occurs. The model accepts an image input, evaluates it on the device, and provides an immediate response with details and disease elimination methods. This was accomplished by training a Siamese neural network model with low computation parameters and operations but high accuracy that can generalize between diverse picture pairs’ class identities. The model is stored on a remote server, where client devices can download it and use it. The mobile app, which is referred to as an Android application, achieved 95% accuracy while just utilizing the device’s resources.

#### Crop disease detection using VGG16, ResNet50, and GoogleLeNet

Anim-Ayeko et al. (2023) proposed a ResNet9 model that detects the blight disease of both potato and tomato leaf images for farmers to leverage. ResNet9 considered leaf shape, its diseased area present, and general green areas for its predictions. The proposed model used PlantVillage dataset of 3990 that were later augmented for training and rigorous hyperparameter optimization procedures. The model was then trained using the hyperparameter settings and tested on a test set of 1331 images. The model achieved 99.25% accuracy, 99.67% precision, 99.33% recall, and an F1-score of 99.33%.

CNN designs were examined by Mohameth et al. (2020), using transfer learning and deep feature extraction, and the generated features were classified using SVM and KNN. Transfer learning attempts to boost the effectiveness of target learners in targeted areas by passing along knowledge from related but separate root domains. Additionally, it is far simpler to build than any CNN design with a randomly determined weight. It is also a quick and efficient approach to utilize features that a previously trained neural network has learned. According to the findings, ResNet50 had the best accuracy of the three when using SVM, whereas GoogLeNet performed better with SVM on the dataset utilized. Using SVM and KNN, VGG16 was the best layer for extracting features for disease classifications. After a classifier was built on top of the layers, SVM outperformed KNN for feature extraction, beating out VGG16 and GoogleNet. VGG16 should be considered for performing transfer learning and fine-tuning a network using a huge dataset. Using the PlantVillage dataset, ResNet50 and VGG16 all achieved accuracy results of 97.82% and 95.3% as results were contrasted using accuracy percentage and execution time.

#### Crop disease detection using CNN

For agricultural disease diagnosis, a solution with an end-to-end Android application built with TFLite and CNN algorithms was presented by Rajendra et al. (2020). In order to efficiently support and aid greenhouse farmers, a DL detection method was employed to identify crop illnesses by photographing the leaves and comparing them to data sets (Rajendra et al. (2020)). Furthermore, users are directed to an e-commerce website where different pesticides, as well as their prices and usage directions, are provided. Visual acquisition and segmentation were used in the process, which sought to make visual display more intelligible and straightforward to assess. Classification was performed by minimizing the sum of squares of distance between the items and their related clusters using a variety of algorithms, with SVM being the recommended method due to its ease of use and reliability. Because the suggested programme detects numerous diseases in a single system, its accuracy was higher than that of CNN.

The project’s goal was to create a tool for better pesticide handling by diagnosing and categorizing plant diseases using contemporary technology and simple internet connectivity Singh et al. (2020). Additionally, a thorough investigation and research on the identification and classification of plant leaves using CNN were provided. For the objective of comprehending the needs in regard to gaps and possible improvements, studies on a variety of conventional ways and current solutions were conducted. Ten thousand (10,000) images from the Kaggle data set were utilized for training and validation. The best architecture produced an accuracy of 96% although other architectures were used as well.

#### Crop disease detection using ResNet34

CNN is thought to be at the cutting edge of image recognition, capable of providing a speedy and accurate diagnosis (Singh et al. (2020)). As a result, the performance of a pre-trained ResNet34 model for crop disease detection is investigated in this study utilizing a web application that can detect seven plant ailments. Singh et al., 2020) refined and implemented a pre-trained CNN that can be accessible online and via a smartphone. For training and validating the model, a dataset of 8685 images taken in a controlled setting was produced. The validation attained 97.2% accuracy, demonstrating the technological viability of CNN in classifying plant diseases. Augmentation and transfer learning aided the model, allowing the CNN to generalize more accurately.

#### Crop disease classification using AlexNet, VGG16, ResNet, and EfficientNet

Tan et al. (2021) intended to find the best ML/DL algorithms for the disease classification issues using the PlantVillage tomato dataset. To manually extract illness features for ML, they used a variety of techniques (a total of 52 texture features utilizing Local Binary Pattern (LBP), GLCM, and feature extraction) (colour). The COLOUR + GLCM technique produced the greatest results out of all. The metrics of the tested DL networks (AlexNet, VGG16, ResNet, EfficientNetB0, and MobileNetV2) (accuracy, precision, recall, and F1-score) were all superior to those of the measured ML techniques (SVM, K-nearest neighbour, and RF). ResNet34 network, using ML/DL algorithms, produced the best results, with 99.7% accuracy, 99.6% precision, 99.7% recall, and a 99.7% F1-score.

An advanced classification model was proposed by Bansal et al. (2021), which detects and classifies tomato leaf disease using CNN (AlexNet for feature extraction and K-nearest neighbour (KNN) for classification). Image feature extraction was used through KNN for classification and AlexNet for feature extraction although the later model achieved 76.1% accuracy that was higher in comparison with KNN and others with a data set for training containing 450 images. Table 4 summarizes the strategies for DL-based crop disease detection and classification.

**Table 4:** Summary methods for DL-based crop disease detection and classification

Reference	Algorithm used	Results	Key contributions	Datasets
Reddy et al. (2023)	DL-CNN, ResNet50, and MRDOA	PDICNet accuracy, 99.73%; precision, 99.83; recall, 99.72; and F1-score, 99.78 ResNet50 accuracy, 99.68%; precision, 99.72%; recall, 99.70%; and F1-score, 99.71	Proposed a customized PDICNet framework/model for crop disease identification and classification	PlantVillage (21,198)
Gandhi et al. (2021)	DL-NN, MobileNetV2, Siamese networks	Accuracy, 95%	Incorporated DL neural networks with MobileNetV2 and Siamese network for reduction of crop loss due to diseases	
Kumbhar et al. (2019)	CNN	Accuracy, 80% training, 89% testing	Proposed a web-based system for cotton leaf disease diagnosis, provide curative and preventative measures	PlantVillage (513) cotton
Anim-Ayeko et al. (2023)	ResNet9	Accuracy, 99.25%; precision, 99.67%; recall, 99.33%; and F1-score, 99.33%	Proposed a ResNet 9 model that detects the blight disease of both potato and tomato leaf images for farmers to leverage	PlantVillage (3990)
Mohameth et al. (2020)	CNN, ResNet50, GoogLeNet, VGG16 SVM, and KNN	Accuracy, 95.3% VGG16, ResNet50 95.38%	The authors evaluated CNN architecture and features obtained classified using SVM and KNN	PlantVillage
Rajendra et al. (2020)	k-means, GLCM, and SVM	Accuracy, 100%	The authors developed an e-commerce website that would compare the pesticide rate and features incorporated into an Android application for detection of the crop disease using CNN	PlantVillage and Private
Singh et al. (2020)	ResNet34	Accuracy, 97.2%	The authors investigated the performance of a pre-trained ResNet34 model in detecting crop disease, which is deployed as a web application	Private (8685)
Singh et al. (2020)	CNN	Accuracy, 97%	The authors created a tool for better pesticide management that would recognize and classify diseases in crops	PlantVillage (10,000)
Tan et al. (2021)	ResNet34	Accuracy, 99.7%; precision, 99.6%; recall, 99.7%; F1-score, 99.7%	The authors compared ML and DL methodology performance and ResNet34 became the best	PlantVillage
Li et al. (2021)	CNN-AlexNet with KNN	Accuracy, 76.1%	The authors proposed an advanced classification model of disease detection and classification	Private
Kumbhar et al. (2019)	CNN	CNN accuracy performance of 80% and 89% on testing	The authors developed a prediction CNN application that would also recognize diseases	Private
Khan et al. (2021a)	CNN, DenseNet-77	Accuracy of 100%	The authors designed a robust crop disease classification framework by introducing a customer CenterNet framework with DensNet 77	PlantVillge
Harakannanavar et al. (2022)	CNN	Accuracy, 94.29%	The authors developed a model using ML for ease of use regarding identification and CNN applications for training and testing	GitHub
Raina and Gupta (2021)	CNN	Accuracy, 97.82%	The authors successfully implemented a new technology for plant disease detection using CNN through Laplacian filter and unsharp masking technique for image processing and canny edge for segmentation	PlantVillage
Rinu and Manjula (2021)	DL-CNN and VGG16	DL_CNN accuracy of 94.8%	The authors designed a system	PlantVillage
Prajwalgowda (2020)	CNN	Incredible performance	Developed a mobile application and availed it for free on the Google Play Store by training the paddy crop disease as well as other crop diseases integration for the purposes of prediction of diseases	Private Paddy

## Discussion

This section discusses the design and development of crop disease detection, classification, and severity assessment techniques focusing on both ML and DL.

### Crop and crop diseases

Crop diseases have been controlled using traditional and mechanical methods, but it has been revealed that these methods are ineffective due to the rapid spread of infections. Because agriculture is the backbone of any nation’s economy, precise and timely disease-related product identification, categorization, and severity testing are required. To do this, timely, rapid, accurate, and cost-effective procedures are required, which is where technological breakthroughs such as artificial intelligence come in.

**Fig. 4:**
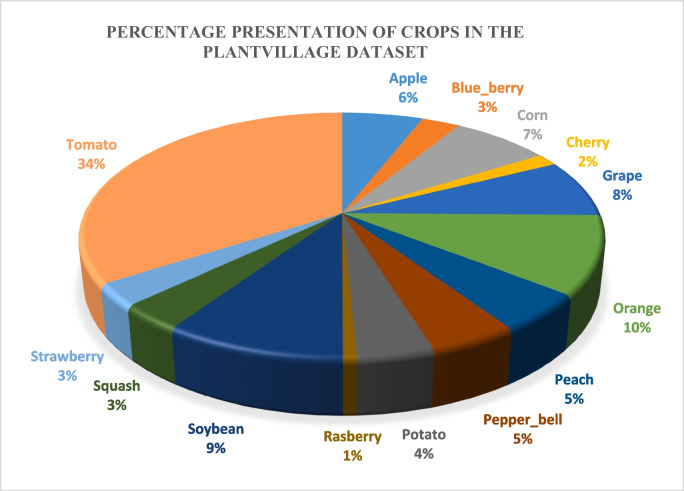
A percentage representation of crop images in PlantVillage dataset

According to the literature study, the majority of studies focused on the following crops including fruits and vegetables: tomatoes, cotton, citrus, rice, cassava, apple, orange, peach, potato, pepper, corn, grape, blueberry, cherry, raspberry, soya beans, squash, and strawberry. Although it appears that most crops, particularly those in the grain, legume, and fruit groups, have not yet been thoroughly investigated, these crops have been discussed by a diverse range of authors. Figure 4 illustrates crop percentages, displaying their popularity through the use of percentages.

Figure 4 shows that the PlantVillage dataset is highly imbalanced due to the significant variation in the number of images among the different classes. Tomato, oranges, and soya bean are overrepresented, while strawberry, raspberry, squash, and cherry are underrepresented. Tomatoes and rice appeared to be the most dominant in crop disease detection and classification.

**Fig. 5:**
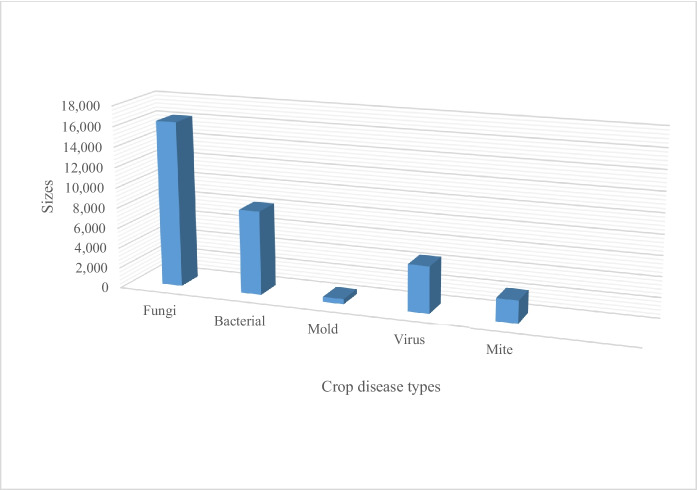
Illustration of crop common diseases as presented in PlantVillage dataset

Additionally, pursuant to the literature review, the majority of studies focused on the following fungi- and bacterial-related crop diseases: rice leaf pests, bacterial blight, brown spot, and leaf smut. As shown in Fig. 5, most studies focused on fungi, bacteria, and virus-related diseases. Few studies focused on mould- and mite-related diseases. This suggests that future studies can concentrate on crop diseases that have not yet been adequately explored, such as groundnuts, green grams, yams, and cashew nuts, to name a few.

### Machine learning-based plant disease detection and classification techniques

ML algorithms are said to be helpful in crop disease detection, classification, and severity estimation evaluation checks. The major aim of using ML techniques is to improve crop disease identification rate as well as the accuracy of the outcome results. This strategy is more real-time, accurate, and time-efficient; also, custom ML-based methods improve decision-making because they produce less precise answers than DL architectures (Harakannanavar et al., 2022). ML applies linear algorithms to either identify or detect crop diseases through feature extraction, with the latter two being the most well-known due to their accuracy in performance results obtained in feature extraction and training.

Kerre and Muchiri (2022) developed a deep CNN model using a novel technique for detecting a simultaneous occurrence of strawberry leaf spot and leaf blight. The detection of these disease classes increases in complexity when they occur on the same leaf. Thus, the importance of the model is to accurately learn the overlapping features in order to detect them. This resulted to the model development with a normalization of the activations of the intermediate layers of the deep neural network. The technique used has an effect of improving model accuracy and speeding up the training process. This was achieved using a private dataset of 1134 strawberry images for both training and evaluation. The model achieved an accuracy of 98%, precision of 89%, recall of 93.3%, and F1-score of 95.9%, thus demonstrating the feasibility of the technique.

**Fig. 6:**
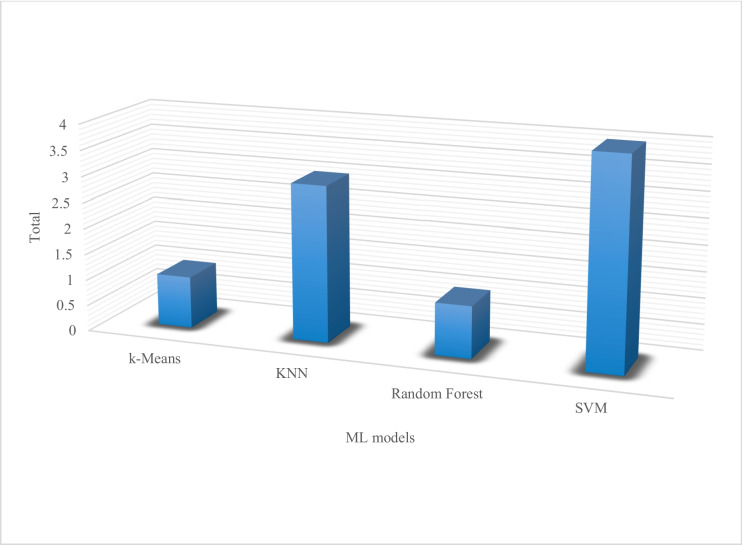
ML algorithm commonly used for crop disease classification and detection

The graph representation in Fig. 6 shows ML’s most commonly used algorithms based on their performance metrics of accuracy in classification and detection of crop diseases. SVM outperforms all others seconded by KNN.

### Deep learning-based plant disease detection, classification, and severity estimation techniques

DL-based techniques have demonstrated cutting-edge accuracy in agriculture and have been successfully extended to a wide range of applications. CNN and ANN are two DL algorithms that have been used in plant disease detection, classification, and severity estimate. Many studies concentrated on CNN, and just a few used current DL algorithms like vision transformers (ViT) and capsule neural network (CapsNet). This could be due to CNN properties like equivalent representations, sparse interactions, and parameter sharing (Kim (2019)). Furthermore, many CNN-based network topologies for plant disease detection, classification, and severity estimate have been employed in the literature. DenseNet, VGG, AlexNet, GoogleLeNet, Inception V3, Inception V4, SqueezeNet, and MobileNet are a few examples of popular network architectures used for crop disease detection and classification.

**Fig. 7:**
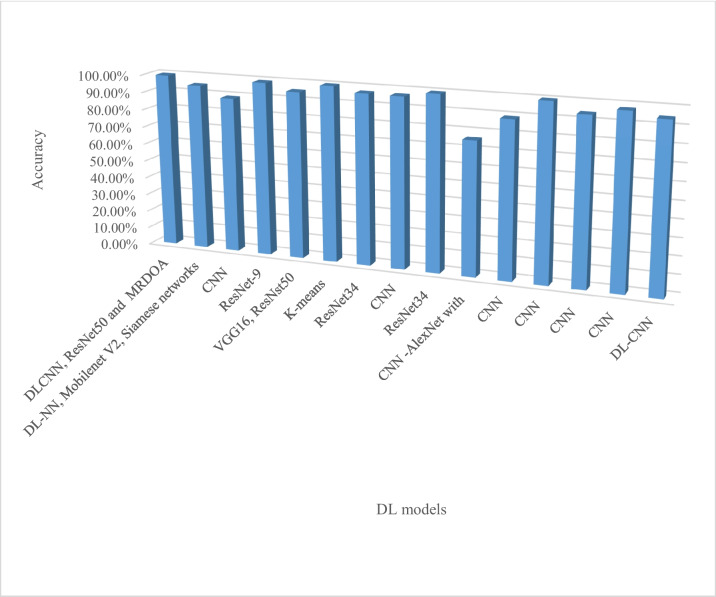
DL Algorithm used in crop disease classification and detection

**Fig. 8:**
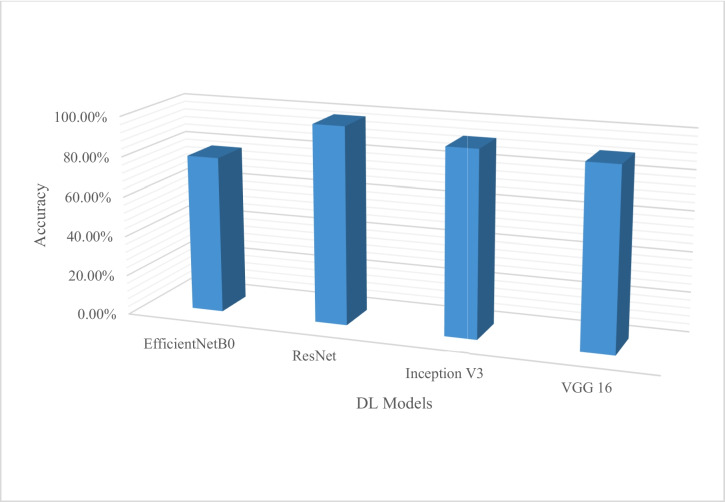
DL algorithm commonly used for crop disease severity estimation

Figure 7 shows a representation of commonly used DL algorithms used in crop disease detection and classification with ResNet and CNN being the core especially on their performance metrics of accuracy. The two can be highly recommended for implementation. Most research used ResNet, VGG, and AlexNet network topologies due to their exceptional performance. Moreover, most research used AlexNet, SqueezeNet, and Inception V3 to estimate disease severity. The DL algorithm commonly used for crop disease severity estimation is presented in Fig. 8.

Nonetheless, crop disease severity estimation seems to be an untapped area of research due to a small number of writers mentioning it, with the majority of authors employing DL approaches and network topologies.

### Datasets

**Fig. 9:**
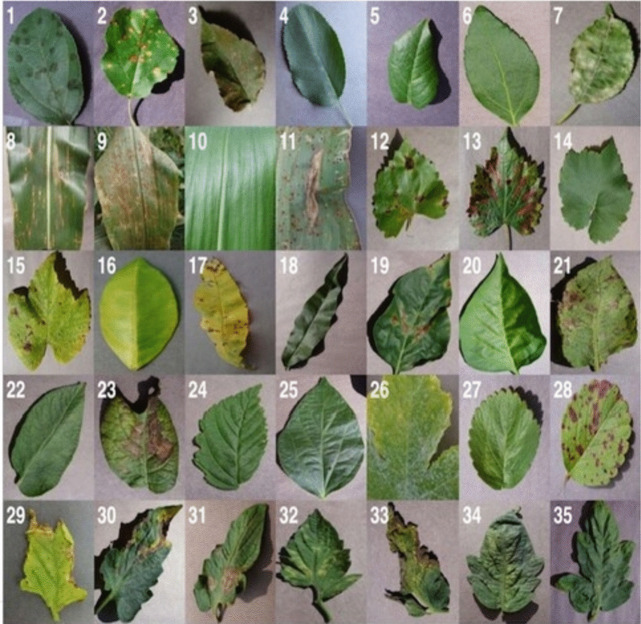
Examples of labelled leaf images from the PlantVillage dataset

Crop dataset frequently comprises high-resolution images of crops shot from various angles and under varied lighting conditions. These images may depict many stages of plant development, such as seedlings, mature plants, and diseased or damaged plants. Additionally, the dataset may contain annotations or labels that provide information about the plant species, plant parts, diseases, or other relevant attributes. As shown in the survey, most studies used the PlantVillage dataset by Hughes and Salathe (2015) whose contents are a collection of images and information related to crop diseases and their symptoms. An example of typical datasets is illustrated in Fig. 9.

In Fig. 9, the 35 labelled images represent every crop-disease pair that can be used for detection and classification purposes within the domain of computer vision. The annotated class names corresponding to each label are detailed in the work presented by Hassan et al. (2021).

**Fig. 10:**
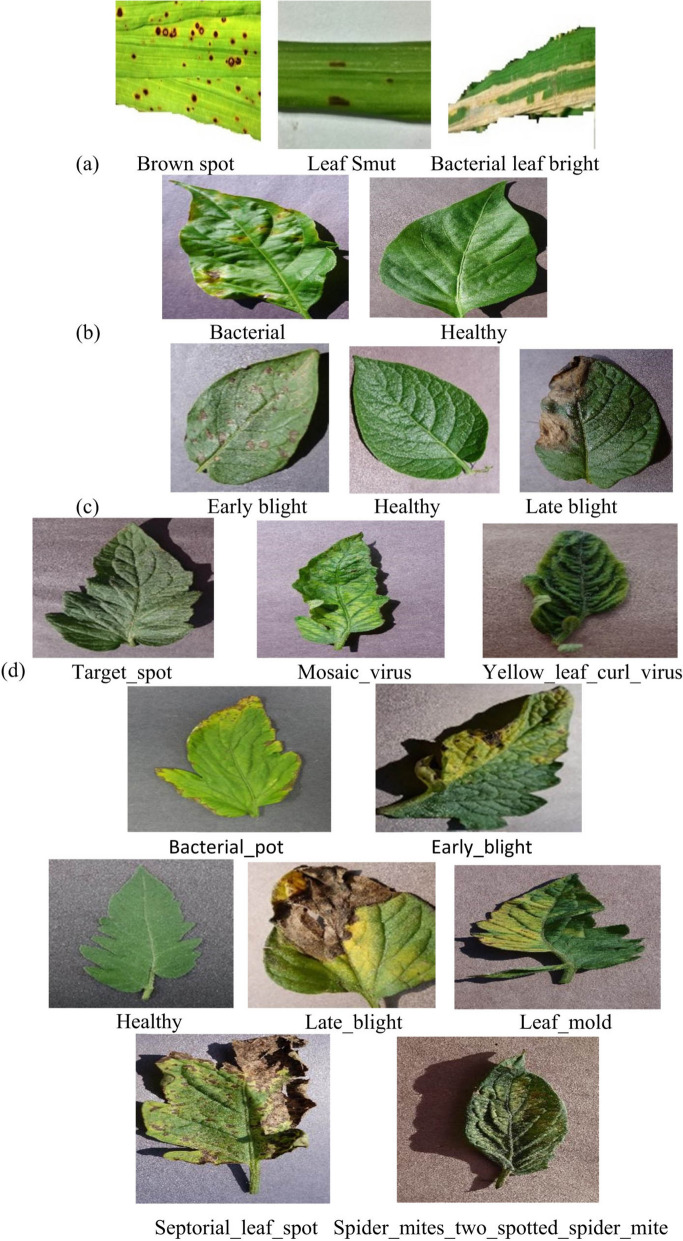
Examples of leaf images illustrating various disease types in the following crops: **a** rice, **b** pepper, **c** potato, and **d** tomato plants

The PlantVillage dataset is a public online platform that provides information and tools to help identify and manage crop diseases and is part and parcel of their effort towards advancing research in this particular field. This dataset contains a wide range of crops including fruits, vegetables, and grains, with a substantial number of labelled images, its purpose being aiding in the development of ML and DL models for automated crop disease diagnosis. Figure 10 illustrates samples of leaf images showing different types of diseases in (a) rice, (b) pepper, (c) potato, and (d) tomato plants (Lamba et al., 2021).

**Fig. 11:**
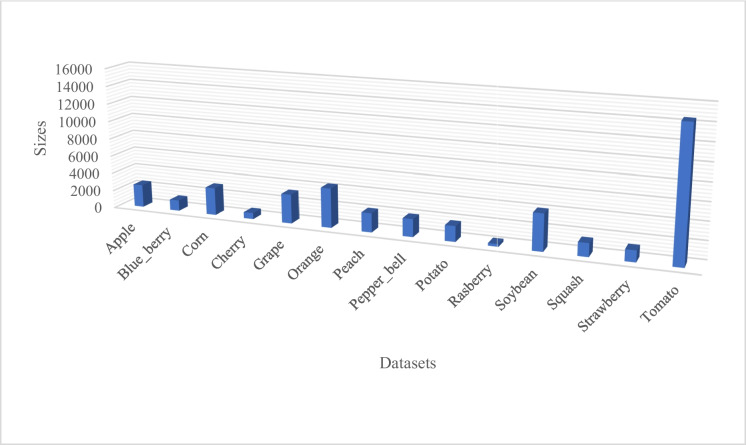
Illustration of different crop images in PlantVillage dataset

The dataset consists of over 54,303 labelled images of both healthy and diseased crop leaves collected under controlled conditions. Specifically, the dataset consists of 14 crop species, including apple, blueberry, cherry, grape, orange, peach, pepper, potato, raspberry, soy, squash, strawberry, and tomato. It also contains 17 basic diseases, 4 bacterial diseases, 2 mould-causing diseases, 2 viral diseases, and 1 mite-caused disease. Twelve crop species also have healthy leaf images that are not visibly affected by disease. Figure 11 shows different crop images that compose the PlantVillage dataset.

### Feature selection

Komala (2021) enhanced the image colour contrast through image pre-processing and segmented the same using k-means segmentation algorithm followed by the GLCM texture feature extractor. The extracted features were later optimally selected to remove redundancy for a better learning and testing process using KNN classifier. Filter method ranking-based technique was also used since it provides independency to the machine learning algorithm as it is of more general approach, less expensive in cost on computation process.

Gajanan et al. (2018) used k-means clustering technique for image segmentation; feature vectors such as image colour, morphology, texture, and structure of hole were applied for extracting features of each image and for disease diagnosis with morphology giving accurate results. Additionally, SURF algorithm which is a pattern matching used to evaluate functions that depend upon a huge number of inputs was used as a locator and descriptor for extracting features that were used to calculate the scope of interest. The algorithm SURF achieved performance matrices of 90% accuracy.

Mohameth et al. (2020) illustrated that the use of feature extraction is a fast and efficient method to take advantages of the features learnt by a pre-trained neural network by propagating the input images to a specified layer that later defines it as an output feature. Hence, the PlantVillage dataset on ResNet50 for deep feature extraction for disease classification using SVM classifier gave an accuracy of 98.0%.

Salih et al. (2020) used convolution operation to extract features such as colour and edges from the image by fixing the size of the filter in all convolution layers with alternating the number of filters. The function of the convolution layers is to extract features such as colour and shape from the inputted image. This feature extraction was used with DL-CNN to detect and classify tomato plant leaf disease.

Batool et al. (2020) used AlexNet in the feature extraction process by applying a 227*227*3 format whereby its middle layer forms the largest part of the AlexNet network. This layer consists of a series of five convolution layers, an activation function ReLu, and a maximum grouping layer where features were extracted. The extracted features were used on KNN classifier for the purposes of crop disease classification which achieved an accuracy of 76.1%.

Tan et al. (2021) extracted colour and texture features from tomato leaf images that were being pre-processed using PlantVillage dataset. The author used haralick texture to represent the image texture by using normalized co-occurrence matrices or GLCM to obtain the distribution of grey values between pixels in a greyscale image to perform feature extraction. The texture was determined based on the number count of pixels. These features were used with Extreme Learning Machine (ELM) classification algorithm with a single-layer feed-forward neural network for crop disease classification.

### Performance overview

Table 5 shows a summary of some DL algorithm performance metrics of accuracy implemented by different authors. Among the best-performing DL algorithm is Inception V3 with an accuracy of 99.72% in the study by Brahimi (2018) who found the possibility of improving the accuracy performance of new CNN architectures.

**Table 5:** Summary of performance metrics of accuracy in DL algorithms used for crop disease detection, classification, and severity estimation

Author (year)	Methodology	Findings	Performance metrics
Brahimi (2018)	Inception V3	Possibility of improving new CNN architectures such as Inception V3 accuracy	99.72% accuracy
Yadav et al. (2021)	VGG16	There is a possibility of training models with other leaf disease images to detect multi-diseases from the peach crops	93.73%
Hassan et al. (2021)	VGG16	The required time for training DL models is much less than that of ML approaches. This is because most of their architectures such as MobileNetV2, an optimized DL-CNN, limit the parameter number and operations as much as possible can easily run on mobile devices	98.87%
Yadav et al. (2021)	VGG16	DL models can be trained with other leaf disease images to detect multi-diseases from the peach crops. Moreover, the models can be installed in the DPS processor to make a standalone device which is portable	98.87%
Batool et al. (2020)	AlexNet	The study achieved 76.1% classification accuracy by using AlexNet model which was found to have the highest performance compared to other models used such as KNN	76.1% accuracy

Arya (2019) showcased DL techniques used to detect crop leaf diseases in a comparative study and analysis of the prominent DL methods. DL uses CNN architecture for object detection since its performance with regard to classification has progressed well in the last few years. Its architecture includes LeNet, AlexNet, GoogleNet, and VGG19 which were used in crop disease detection using both PlantVillage dataset and private dataset. AlexNet by observation was the most frequently used. Inception V3 and ResNet34 showed 99.72% and 99.67% performance accuracy with VGG13 99.49% while used with DL transfer strategy (Brahimi, 2018).

**Fig. 12:**
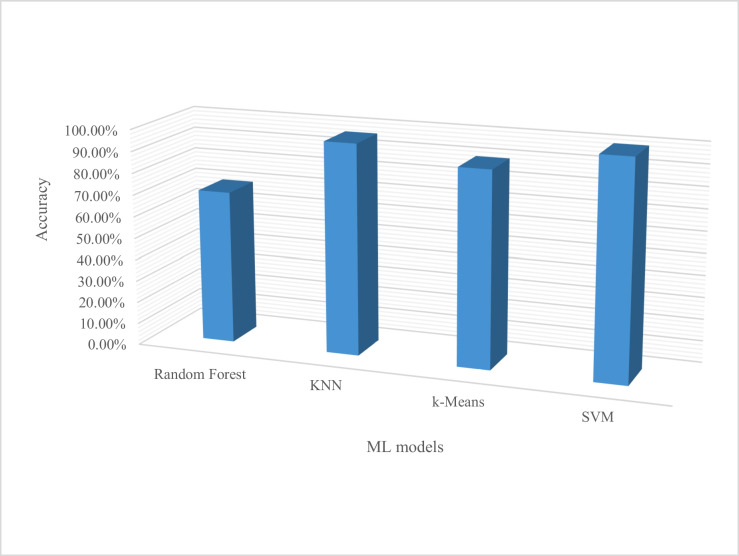
ML algorithms accuracy performance used for crop disease detection and classification

In Yadav et al.’s (2021) identification of disease using DL and evaluation of bacteriosis in peach leaf, VGG16 showed an accuracy performance of 93.75%. Hassan et al. (2021) in plant leaf disease identification using CNN and transfer-learning approach indicated an accuracy performance of 98.87% in VGG. Other studies highlighted other algorithms used in ML for crop disease classification and detection with SVM that achieved an accuracy performance of 100% and KNN with 97% accuracy. The two classifiers are being preferred, due to their standard of performance from the analysis represented in Fig. 12.

**Fig. 13:**
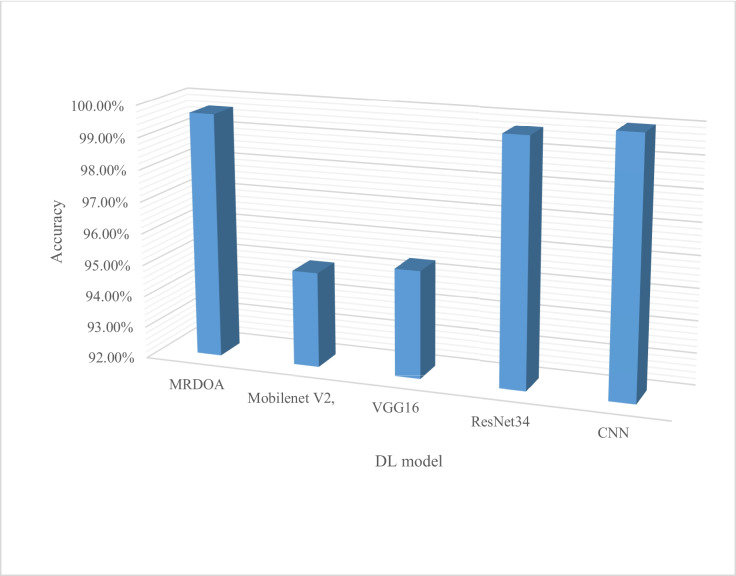
DL algorithm commonly used for crop disease classification and detection

DL’s most commonly used algorithm was CNN with 100% accuracy and ResNet with 99.7% accuracy due to their commendable performance metrics of accuracy as shown in Fig. 13.

In summary, the presented review tasks have explored deep into the various applications of some significant cutting-edge ML and DL algorithms in the research field of crop disease detection, classification, and severity estimation. It highlights the transition from traditional methods to advanced deep learning techniques, emphasizing the advantages in accuracy and efficiency they bring to agricultural technology. The review examines performance analysis methodologies for the latest machine learning and deep learning algorithms, explores the impact of environmental factors on dataset quality, and encourages the use of versatile sensors for clearer image data. It also recommends broader research coverage encompassing a wider array of crops and diseases, the fusion of multiple state-of-the-art technologies, and a more holistic approach to crop disease management. Additionally, the review identifies the need for more precise CNN architectures, explores data collection at various disease stages, and suggests the incorporation of robotic features. Overall, this review serves as a roadmap for future advancements in the field of crop disease management, emphasizing the transformative potential of deep learning technology.

### Prospects, challenges, and future work

The prospects of machine learning and deep learning approaches in the domain of crop disease detection, classification, and severity estimation are highly promising. These advanced technologies offer a range of benefits and hold significant potential for the future of agriculture. Several literature have equally expanded on some of the significant contributions and benefits that can be derived from the potential application of ML and DL algorithms (Diseases, 2017; Ghosal et al., 2018; Kamilaris & Prenafeta-Boldú, 2018; Sladojevic et al., 2016)

According to existing studies (Kamilaris & Prenafeta-Boldú, 2018; Mohanty et al., 2016) and findings from the current study, several authors have shown that robust ML and DL techniques have the capabilities of identifying subtle symptoms of diseases in crops well before they become visible to the eye, and this early detection can indeed provide handy information to the farmers, thereby enabling them to take specific proactive measures for timely intervention that can lead to optimal reduction in crop losses. Another obvious advantage which has been projected by these robust learning tools is their inherent potential to generate high-level accuracy in disease classification and severity estimation, ensuring that the right treatment is applied to the affected crops.

It is of paramount importance to highlight that the automation of disease detection processes can significantly reduce the workload on farmers and agricultural experts. It allows for the monitoring of large agricultural areas, making it more cost-effective and efficient. Another positive factor of incorporating ML and DL for the purpose of early detection of crop disease includes the fact that these intelligent models can be tailored to specific crops, diseases, and environmental conditions, making them adaptable to various agricultural scenarios. More so, by integrating these interesting technologies with Internet of Things smart devices such as sensors and drones would enable real-time monitoring of crops, thereby allowing for immediate responses to disease outbreaks. However, there are challenges to address as well, including data quality, model interpretability, data privacy, and the need for continued research to improve the robustness and adaptability of these models. Next, we present a summary of the key challenges facing the deployment of ML and DL technology for the tasks of crop disease management (Barbedo, 2018; Matin et al., 2020; Wani et al., 2022). These challenges are highlighted as follows:

i. Generalization to diverse environmental conditions: One of the key challenges is developing deep learning models that can generalize well across various environmental conditions and lighting scenarios, ensuring robust performance under real-world, unpredictable circumstances.
ii. Data quality and diversity: Obtaining high-quality, diverse datasets that encompass different crop types, geographical regions, and stages of disease development remains a persistent challenge. Ensuring that these datasets are representative of real-world conditions is crucial.
iii. Reducing data annotation requirements: Deep learning models often demand extensive data annotation efforts. Finding ways to minimize the labelling burden, such as through semi-supervised learning or active learning, is an ongoing challenge.
iv. Interoperability and integration: Integrating deep learning systems into existing agricultural workflows and equipment can be complex. Ensuring interoperability with various hardware and software platforms remains a challenge.
v. Real-time processing: Achieving real-time or near-real-time processing for crop disease detection in the field, especially on resource-constrained devices, is a significant technical challenge that requires optimization and model compression.
vi. Continual learning and adaptation: Developing models that can continually adapt to evolving disease strains and environmental changes is vital for sustainable crop management. Implementing lifelong learning and adaptation mechanisms is an ongoing research challenge.
vii. Ethical and privacy concerns: As deep learning systems become more integrated into agriculture, concerns about data privacy, ownership, and ethics need to be addressed. Balancing technological advancements with ethical considerations is a growing challenge.
viii. Transferability across crops: Ensuring that deep learning models developed for one crop can be adapted and transferred to others is a challenge, especially when dealing with variations in plant structures, diseases, and growth patterns.
ix. Interdisciplinary collaboration: Bridging the gap between deep learning experts and agricultural domain specialists is an ongoing challenge. Effective interdisciplinary collaboration is essential for developing practical solutions.
x. Validation and interpretability: Establishing robust validation procedures for deep learning models and improving their interpretability are critical challenges to gain trust from end-users and regulatory bodies.

We believe from this extensive study that addressing the aforementioned open challenges will be crucial in harnessing the full potential of these state-of-the-art deep learning techniques for crop disease detection, classification, and severity estimation in agriculture.

Given the profound significance of machine learning and deep learning methodologies, which are at the forefront of transforming the complexities associated with crop disease detection, classification, and severity estimation, the key takeaway from our research is the optimistic belief that through ongoing progress and interdisciplinary cooperation, the persistent utilization of ML and DL technologies will undoubtedly serve as a central factor in safeguarding worldwide food security and fostering sustainable agricultural practices. The following recommendations, stemming from the insights of the present study, offer potential avenues for advancing research in the realm of applying ML and DL to crop disease management. These suggestions encompass the following:

i. Significantly enhancing the efficiency of convolutional neural networks (CNN), which typically comprise three layers, including convolution, max-pooling, and complex fully connected layers. The latter is computationally intensive and memory-consuming, necessitating the exploration of more memory-efficient and computationally intricate technologies.
ii. Addressing the issue of environmental factors and background data that often affect the clarity of images in datasets. Exploring the use of versatile sensors, such as infrared and multispectral cameras, could yield clearer, noise-free images, thereby improving diagnostic accuracy.
iii. Expanding the scope of research beyond the examination of just two crops, each with a limited number of diseases. Consideration should be given to studies encompassing more diverse crops, each with a broader range of diseases.
iv. Recognizing the potential of combining multiple state-of-the-art technologies, a practice that is still relatively rare. Instances where deep learning (DL) and machine learning (ML) classifiers were combined have shown commendable results in terms of performance and accuracy, suggesting that further exploration of this fusion may yield improved outcomes.
v. Balancing the focus on crop disease classification and detection with a greater emphasis on disease severity estimation. The potential of employing an ensemble framework that integrates all three aspects—disease detection, classification, and severity estimation—should be explored, as it offers a comprehensive approach to managing crop diseases.
vi. Collecting data that spans different stages of crop diseases, facilitating the design of algorithms tailored to crop disease severity estimation, ultimately leading to more accurate diagnoses and treatment.
vii. Investigating various factors influencing the selection and optimization of DL architectures, including data augmentation techniques, batch size, and epoch configurations. Additionally, exploring the integration of robotic features for enhanced crop disease detection, classification, and severity estimation can yield more effective outcomes.
viii. Developing more accurate CNN architecture algorithms that combine three state-of-the-art pre-trained models (GoogLeNet, VGG16, ResNet, DenseNet). Pre-processing, segmentation, and classification of data using these algorithms can lead to more precise detection, classification, and severity estimation of crop diseases.
ix. Expanding research beyond the examination of crop leaves to include other plant parts, such as roots, stems, branches, and more, to create a more holistic approach to disease diagnosis and management.

## Conclusions

In conclusion, our comprehensive review of state-of-the-art deep learning approaches for crop disease detection, classification, and severity estimation has shed light on the evolving landscape of agricultural technology. The advances in deep learning have revolutionized our ability to tackle the pressing challenges of plant disease management. Through our exploration of contemporary literature, we have identified a significant shift from traditional methodologies to the adoption of deep learning techniques. This transition has not only enabled more accurate and efficient crop disease diagnosis but has also facilitated early detection, thereby aiding in timely interventions and crop protection.

The performance analyses of the latest machine learning and deep learning algorithms showcased in our review have highlighted their potential and provided valuable insights for researchers and practitioners. It is evident that the power of CNN, RNN, CapsNet, and ViT has been harnessed to create innovative solutions, pushing the boundaries of crop disease management. In addition, our discussion of publicly available plant datasets emphasizes the need for collaborative efforts in data collection and sharing, ensuring that researchers have access to diverse and representative data, which is essential for training robust deep learning models.

As we consider the recommendations and gaps identified in the reviewed literature, it becomes apparent that there is still much work to be done. To advance this field further, we propose the development of a unified framework that integrates a variety of deep learning and machine learning algorithms to address the complexities of multiple plant diseases effectively. Moreover, the creation of a comprehensive and diverse image dataset, encompassing a wider array of crop types and diseases, remains a priority.

It is equally important to highlight from the concluded study that the application of state-of-the-art deep learning techniques in crop disease detection and management is a promising and dynamic field. It holds the potential to significantly impact global agriculture by enhancing disease control, reducing crop losses, and ultimately contributing to food security. This review serves as a testament to the remarkable progress made in recent years and encourages continued exploration and innovation in the realm of agricultural technology.

## Data Availability

Data is available from the authors upon reasonable request.
